# Arteriovenous Blood Metabolomics: A Readout of Intra-Tissue Metabostasis

**DOI:** 10.1038/srep12757

**Published:** 2015-08-05

**Authors:** Julijana Ivanisevic, Darlene Elias, Hiroshi Deguchi, Patricia M. Averell, Michael Kurczy, Caroline H. Johnson, Ralf Tautenhahn, Zhengjiang Zhu, Jeramie Watrous, Mohit Jain, John Griffin, Gary J. Patti, Gary Siuzdak

**Affiliations:** 1 Center for Metabolomics and Mass Spectrometry, The Scripps Research Institute, 10550 North Torrey Pines Road, La Jolla, California 92037, United States; 2 Department of Molecular and Experimental Medicine, The Scripps Research Institute, 10550 North Torrey Pines Road, MEM-180, La Jolla, California 92037, United States; 3 Anticoagulation Services, Scripps Clinic and Scripps Green Hospital, 10666 North Torrey Pines Road, W207, La Jolla, California 92037, United States; 4 Departments of Medicine and Pharmacology, University of California, 9500 Gilman Drive, MC 0613, La Jolla, California 92037, United States; 5 Departments of Chemistry, Genetics, and Medicine, Washington University, One Brookings Drive, St. Louis, Missouri 63130, United States; 6 Departments of Chemistry, Molecular and Computational Biology, The Scripps Research Institute, 10550 North Torrey Pines Road, La Jolla, California 92037, United States

## Abstract

The human circulatory system consists of arterial blood that delivers nutrients to tissues, and venous blood that removes the metabolic by-products. Although it is well established that arterial blood generally has higher concentrations of glucose and oxygen relative to venous blood, a comprehensive biochemical characterization of arteriovenous differences has not yet been reported. Here we apply cutting-edge, mass spectrometry-based metabolomic technologies to provide a global characterization of metabolites that vary in concentration between the arterial and venous blood of human patients. Global profiling of paired arterial and venous plasma from 20 healthy individuals, followed up by targeted analysis made it possible to measure subtle (<2 fold), yet highly statistically significant and physiologically important differences in water soluble human plasma metabolome. While we detected changes in lactic acid, alanine, glutamine, and glutamate as expected from skeletal muscle activity, a number of unanticipated metabolites were also determined to be significantly altered including Krebs cycle intermediates, amino acids that have not been previously implicated in transport, and a few oxidized fatty acids. This study provides the most comprehensive assessment of metabolic changes in the blood during circulation to date and suggests that such profiling approach may offer new insights into organ homeostasis and organ specific pathology.

Arterial and venous blood circulation represents the principle integrating system of the human anatomy, serving to maintain homeostasis in all tissue interstitial fluids. The preservation of homeostasis depends primarily upon the biochemical correspondence between the cells and arterial and venous blood, where the essential vectors and mediators are metabolites. This “metabostasis” is crucial for optimal cell function and intercellular communication and cooperation^1^. Blood is a primary carrier of metabolites including a variety of nutrients (carbohydrates, lipids and amino acids), hormones, electrolytes, metabolic by-products and organic wastes. The acknowledged concept of arterial blood supplying the tissues with nutrients (and oxygen) and venous blood carrying the metabolic by-products (and carbon dioxide) from tissues and organs is one of the best examples of metabolite transport *in vivo*^2^,^3^. The uptake of nutrients by metabolizing tissues and release of by-products is clearly reflected in oxygen consumption as basal arteriovenous difference for oxygen content (~40 mL/L). Beyond measurements of oxygen consumption coupled to the measurement of glucose uptake and early amino acid balance studies across major organs^2^,^3^,^4^,^5^,^6^, surprisingly little research has been performed on metabolic blood homeostasis at different levels of the circulatory system. Since these early, fundamental metabolism studies, performed between 1930’s and 1970’s, when the available analytical methods allowed for the analysis of only a limited number of metabolites, the arterio-venous approach of simultaneous sampling of arterial and venous blood has been markedly discontinued. Few follow up studies in the 1990’s required several different analytical techniques, including spectrophotometry, spectrofluorimetry, ion-exchange chromatography and enzymatic assays to target the metabolites of interest^7^,^8^,^9^. Technology, specifically mass spectrometry, has since improved by orders of magnitude in terms of sensitivity, accuracy, dynamic range and ability to observe intact biomolecules, thereby offering many new insights into metabolic processes and organismal biology^10^,^11^,^12^,^13^.

Technology-driven metabolomic research in clinical diagnostics is evolving rapidly and blood is one of the most widely used biofluids for metabolic phenotyping^14^,^15^,^16^. Targeted mass spectrometry blood assays are already applied in clinical research diagnostics, in screening for congenital, inborn errors of metabolism or acquired metabolic disorders^17^,^18^,^19^,^20^. While targeted metabolomics is a hypothesis driven approach that screens for a defined set of known, potential biomarkers of disease, untargeted metabolomics is a discovery-oriented approach, designed to comprehensively measure metabolite pools within an organism and generate hypothesis about unanticipated changes in metabolism^10^. The majority of untargeted, blood metabolomic studies today focuses on disease biomarker discovery by profiling exclusively venous blood^15^,^21^,^22^,^23^. Due to high inter-individual variability of human metabolic profiles (which fluctuate markedly depending on age, gender, diet, drug therapy and other aspects of lifestyle) it is difficult to determine whether the subtle variations in concentrations of metabolites, measured only in venous blood, are related to a specific disease and/or side effects from a particular drug. While more easily accessible, venous blood reflects the metabolism of the particular organ drained^6^, and can thus yield a biased profile of the body’s metabolic status. In contrast, arterial blood represents the fluid prior to being processed by any particular organ, reflecting better the average metabolic state of the body^24^.

To extend the value of blood metabolite profiling beyond biomarker discovery, we performed global profiling of paired arterial *vs.* venous human blood at the skeletal muscle level of a circulatory system to asses metabolic changes in the blood during circulation in a more comprehensive manner. A recently developed, hydrophilic interaction chromatography-mass spectrometry (HILIC/MS) based untargeted metabolomic approach^12^ has enabled us to screen several thousand metabolite features present in the plasma metabolome. The metabolite levels that were initially revealed as changing in arterial *vs.* venous plasma were further measured, together with their counterparts from related biochemical pathways, using a targeted approach. The respective, subtle, yet highly significant changes in specific metabolite levels in arterial *vs.* venous plasma are discussed in the light of the biochemical nutrient cycling and energy gradients. We introduce the concept of global arteriovenous metabostasis analysis to study intra-tissue metabolic activity and gain insights into normal organ homeostasis and potentially organ specific pathology, by simultaneously assaying the broad range of chemically diverse metabolites.

## Results

### Global Metabolite Profiling Reveals Changes in Arterial *vs.* Venous Human Blood during Circulation

**Figure 1:**
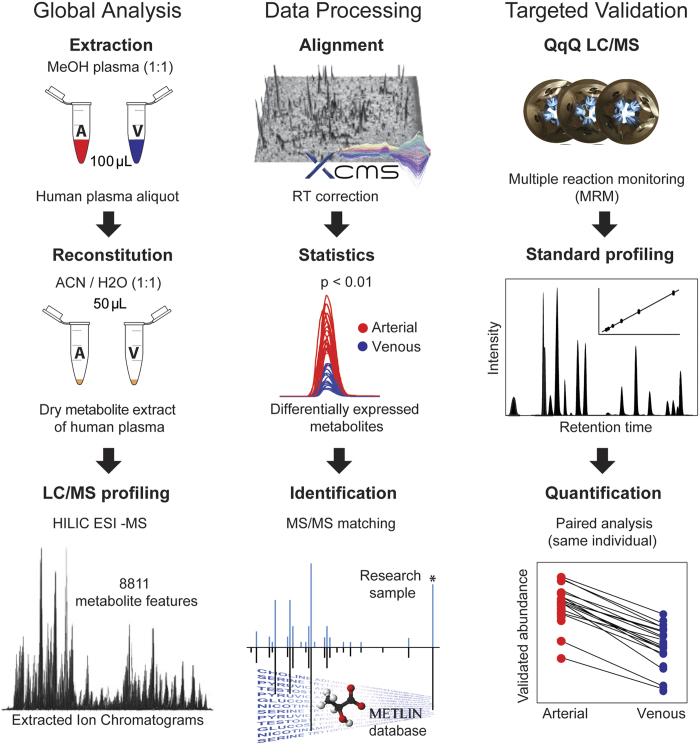
Three consecutive steps in metabolomic workflow. Global profiling summarizes the experimental design including metabolite extraction and reconstitution protocol prior to untargeted HILIC/MS profiling in ESI negative mode. LC/MS data acquisition was followed by retention time correction and chromatogram alignment. Metabolite features whose levels significantly changed (p < 0.01) in venous *vs.* arterial plasma were filtered out and identified by MS/MS matching. The identified metabolites were quantified by targeted MRM analysis using standard compounds. All drawings were created by authors except the Eppendorf schemes that were downloaded as a vector clip art from the website www.clker.com.

**Figure 2:**
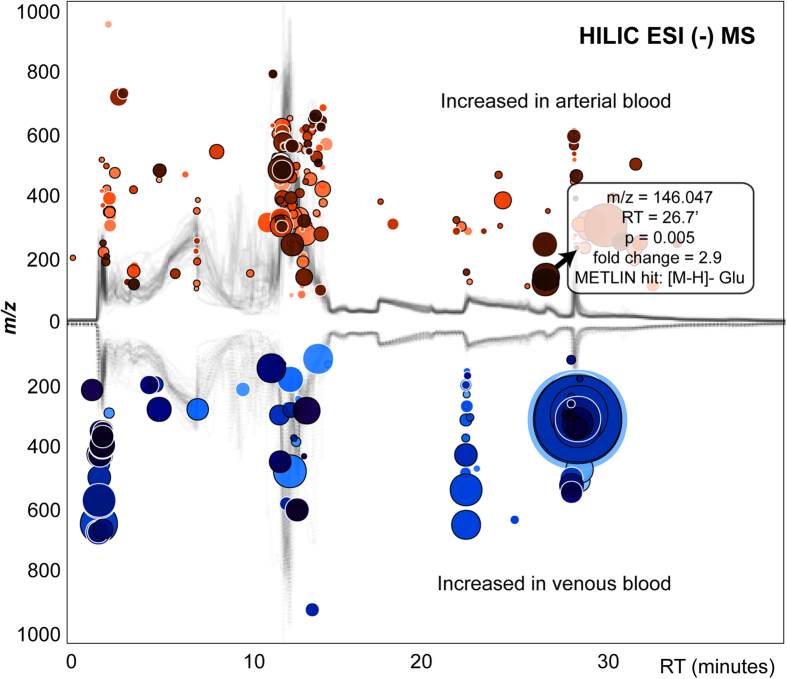
Cloud plot showing metabolite features (represented by “bubbles”) whose levels vary significantly between arterial and venous blood (p < 0.01, Int > 10,000, median fold change > 1.3). The fold change is used as radius scale of each bubble. Darker color (in red and/or in blue tones) of the bubble indicates lower p-value.

**Figure 3:**
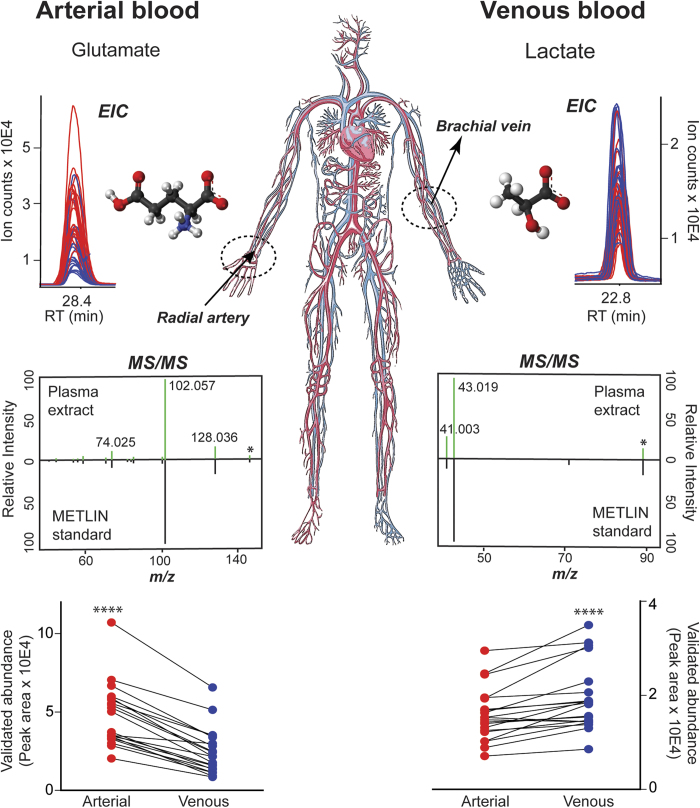
Simultaneous measurement of metabolite levels in human arterial and venous blood. Two key gluconeogenic metabolites whose levels changed significantly after the blood passage through the forearm skeletal muscle. The changes revealed by untargeted metabolite profiling (p < 0.0001, Wilcoxon matched pairs rank test) were identified as glutamate and lactate, respectively, by MS/MS matching against standards in the METLIN database and validated by targeted analysis (paired plots). EIC—Extracted Ion Chromatogram. MS/MS—Tandem mass spectra or fragmentation pattern. Four stars—p value < 0.0001. Michael Kurczy and Julijana Ivanisevic have illustrated the blood circulation using Adobe Illustrator.

Untargeted metabolite profiling was performed initially to examine the differences between arterial and venous plasma across human forearm tissue, collected from healthy adult individuals (female:male 1:1) at rest, after an overnight fast. The population cohort description, including the gender, age, height, weight and body mass index, is given in the Supplemental Table S2. The approach using hydrophilic interaction chromatography coupled with negative ionization mode mass spectrometry based on 80% methanol extraction was applied to maximize the coverage of central carbon metabolites in human plasma (Fig. 1). The analytical strategy using HILIC in negative ionization mode enabled the detection of 8811 metabolite features in the water soluble and lipid plasma metabolome. Overall, untargeted profiling of arterial and venous plasma revealed high inter-individual variability with specific metabolic phenotypes characterizing each individual. These personalized metabotypes may interfere with profile alignment and comparisons, therefore paired analysis of the arterial and venous blood were essential when conducting global (untargeted) profiling of human blood. Many nutrients and metabolic by-products transported in plasma were analyzed across all subjects, including amino and non-amino organic acids, purines and pyrimidines, endogenous sugars, fatty acids, and dietary metabolites and their breakdown products (e.g. tagatose, paraxanthine). The global profiling followed by comparison of paired metabolite levels (measured simultaneously in the same individual) highlighted the significant changes (p < 0.01) in venous *vs.* arterial plasma during circulation across the human peripheral tissue, precisely the skeletal muscle (Fig. 2). Characteristic metabolites with significantly different levels in venous *vs.* arterial plasma were filtered out using the interactive XCMS Online platform and identified using MS/MS pattern matching against METLIN - the largest standard metabolite database (Fig. 1)^25^,^26^. Among significantly changing metabolites, several metabolites involved in gluconeogenesis body cycle displayed highly consistent ratios between arterial and venous levels. Glutamate showed a significant decrease in venous *vs.* arterial plasma (p = 3.8E-06, median fold change = 2.9, Fig. 3) across all 20 subjects. Lactate demonstrated consistent difference characterized by significant increase in venous plasma (p = 4.2E-04, median fold change = 1.4, Fig. 3). In addition, two variants of oxidized unsaturated fatty acids, putatively identified as hydroxyperoxy octadecadienoic acids (HPODEs), were found highly increased in venous plasma ([M-H]^−^ = 311.2224 and [M-H]^−^ = 309.2067, with p = 9.7E-04 and 4.2E-04, respectively, and a median fold change > 30). The exact structure of these specific metabolites was not confirmed by further analysis, due to many potential variants and the absence of MS/MS matching hits in available databases (METLIN, HMDB, and LIPID MAPS)^27^,^28^,^29^.

The putatively identified metabolites whose levels were found to significantly differ in arterial and venous plasma by global profiling (listed in Supplementary Table S1) were quantified by triple-quadrupole (QqQ)-based targeted analysis to validate the results and eliminate potential false positives (see workflow in Fig. 1). Targeted analyses were multiple-pathway focused in order to measure many additional intermediates, associated with the metabolites highlighted by untargeted analysis. Numerous amino acids (21) and other non-amino organic acids, playing an important role in carbon and nitrogen shuttle through glycolysis and gluconeogenesis were targeted together with glutamate and lactate. To interrogate differences highlighted by untargeted analysis further, targeted quantification was also expanded to include tricarboxylic acid (TCA) cycle intermediates, the intermediates of purine and of phenylalanine metabolism.

### Multiple Pathway Targeted Analysis to Elucidate the Metabolite Transport at the Skeletal Muscle Level of the Circulatory System

**Figure 4:**
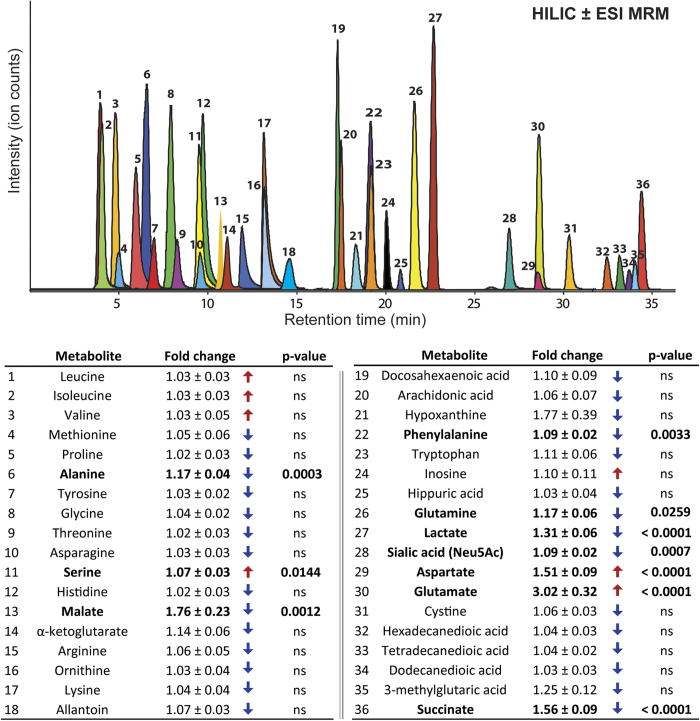
Targeted multiple reaction monitoring (MRM) of 36 endogenous metabolites in human arterial and venous plasma. Extracted ion chromatograms (EICs) represent the quantifier transition ions for each quantified metabolite. Metabolites were quantified by three different methods specified in Methods Section. Mean arteriovenous fold change (A/V or V/A ratio) across 20 subjects (Mean ± SEM) is indicated in the table. Direction of the fold change is illustrated by colored arrows: red arrows – UP in arterial blood (positive arteriovenous balance = taken up by the organ = lower in venous blood) and blue arrows – UP in venous blood (negative arteriovenous balance = released by the organ). Significant metabolite changes (Wilcoxon matched pairs rank test) are indicated in bold.

**Figure 5:**
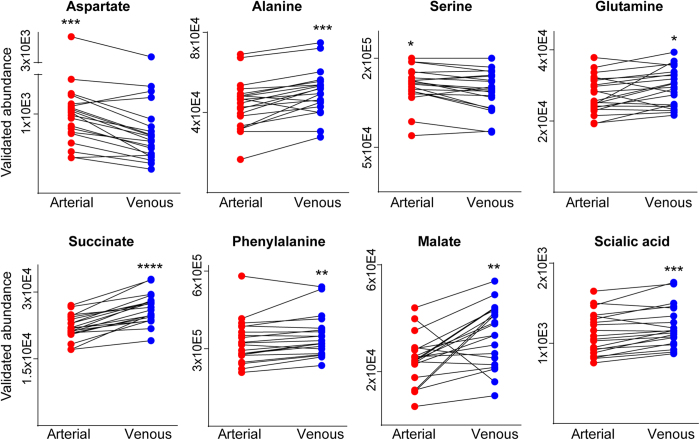
Metabolites that varied significantly after the blood circulation across the muscle tissue, as validated by targeted analysis. Paired plots show the abundances of each metabolite in arterial and venous plasma of each individual. Significance level of Wilcoxon test is indicated by the number of stars.

Changes in arterial *vs.* venous levels were determined for 36 endogenous metabolites using targeted multiple reaction monitoring. The summary of quantified metabolites with the associated arteriovenous fold changes (arterial/venous (A/V) or venous/arterial (V/A) ratios) and levels of significance is given in Fig. 4. Direction of the fold change implies either the positive arteriovenous balance reflecting the metabolite uptake by the organ or the negative arteriovenous balance reflecting the metabolite release by the organ. Metabolite levels (i.e. dynamic range) in both, arterial and venous plasma, varied significantly from one individual to another, up to 10 times, depending on the metabolite of interest. Lactate, was measured together with glucose, as the most abundant among the quantified metabolites (1453 ± 109.4 μM in arterial and 1728 ± 180.1 μM in venous plasma, Figure S2), followed by amino acids (glutamine, glutamate) also detected in high concentrations (≥100 μM) in arterial and venous plasma (Figure S2). Levels of ten different amino and non-amino organic acids were observed to change significantly between arterial and venous plasma, following the passage through the human peripheral tissue (skeletal muscle). Although subtle, the arteriovenous fold changes (from V/A = 1.09 fold for sialic acid to A/V = 3.02 fold for glutamate, Fig. 4, Figure S1) were consistent across the majority of analyzed subjects (Fig. 5). Glutamate showed the highest uptake pattern (or the highest positive arteriovenous balance (3.02 ± 0.32 fold lower in venous plasma, p < 0.0001, Figs 3 and 4) while lactate (1.31 ± 0.06 fold higher in venous plasma, p < 0.0001, Figs 3 and 4) and succinate (1.56 ± 0.09 fold higher in venous plasma, p < 0.0001, Figure S1) displayed the most significant release pattern across human forearm tissue (Fig. 4). In addition to glutamate; aspartate and serine displayed consistent uptake patterns as well, with significantly decreased levels in venous *vs.* arterial plasma while alanine, glutamine and phenylalanine together with lactate, succinate, malate and sialic acid exhibited a consistent release pattern with significantly increased levels in venous *vs.* arterial plasma (Figs 4 and 5). The majority of other targeted amino acids exhibited higher content in venous plasma (qualified as negative arteriovenous balance, e.g. tryptophan, cystine, arginine, aspargine, glycine, histidine, ornithine, Fig. 4), although the difference was not statistically significant. The exceptions were branched amino acids (BAA: leucine, isoleucine and valine), which displayed a positive arteriovenous balance (like previously mentioned glutamate, aspartate and serine) although the statistical significance for BAA was not reached (Fig. 4).

## Discussion

This study was designed to gain a comprehensive and quantitative overview of metabolite transport, reflected in nutrient uptake from arterial blood and metabolic product release into venous blood^3^,^25^, at the specific level of the circulatory system. In contrast to traditional venous plasma screening, metabolite profiling in paired arterial and venous plasma of the same individual was essential to cope with high variability of human biofluid profiles. Moreover, the paired arteriovenous profiling provided an insight into the metabolic activity of a particular tissue. Here we profiled arterial and venous plasma across human forearm tissue where skeletal muscle appears the predominant contributor to the observed metabolic balance or metabostasis. The untargeted metabolomic approach, using hydrophilic chromatography in negative ionization mode allowed for detection of broad set of central carbon and lipid metabolites in human plasma. The results implied the dominant role of central carbon metabolites in the metabolite transport across human skeletal muscle and highlighted the importance of profiling highly polar metabolites, which cannot be retained by reversed phase analyses^12^,^30^,^31^. Overall, the metabolites that varied between arterial and venous blood showed highly significant differences (p ≤ 0.01) yet subtle fold changes (A/V or V/A) ranging from 1.1 to 3. These observations are in accordance with the results from early amino acid metabolism studies that revealed small, although physiologically important arteriovenous differences at different points in the body^6^,^32^. It is important to note that while the untargeted metabolite profiling pointed out the arteriovenous differences and related biochemical pathways, the follow up (“*post-hoc*”) targeted analyses were essential to validate the observed changes and quantify the additional intermediates. Targeted metabolite profiling eliminated the bias due to complex matrix effects (e.g. ion suppression) present in untargeted analysis, effectively eliminating false positives^10^,^33^.

**Figure 6:**
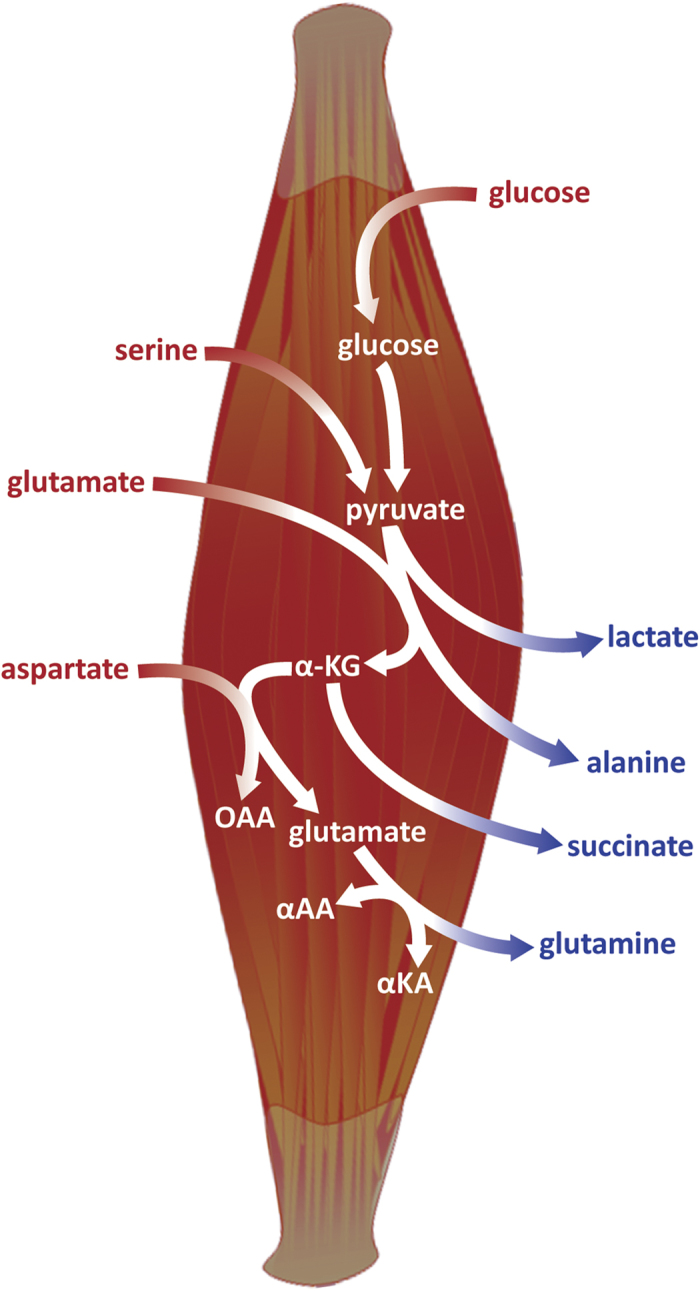
Simplified scheme of main biochemical transformations taking place at the skeletal muscle level of the circulatory system. The observed significant arteriovenous metabolite changes that fit into the context are pointed out in red (glucose, glutamate, aspartate, serine) and blue (lactate, alanine, glutamine and succinate). The uptake pattern of glucose, glutamate, aspartate and serine (in addition to being formed by breakdown of muscle proteins) demonstrates their use by muscle cells as supply for TCA cycle as well as nitrogen and/or carbon donors in the synthesis of alanine, glutamine and other amino acids. Lactate, alanine, glutamine and succinate produced by muscle cells are released into the venous blood. α-KG—α-ketoglutarate, OAA—oxaloacetate, αAA—α-aminoacids, αKA—α-ketoacids. Drawing of a muscle has been downloaded from Science Slides for MS PowerPoint (VisiScience Corporation).

The differences in arterial *vs.* venous plasma, primarily observed in amino acid and other organic acid levels, appear to mainly reflect the metabolic requirements of muscle tissue in a postabsorptive state, the period following an overnight fast when the blood is usually sampled in the process of clinical examination. The postabsorptive metabolic state in muscle is primarily associated with an increase in glycolysis, resulting in an excess of pyruvate^7^,^34^. Our observations indicate that the excess of pyruvate independently governs the production of lactate, and the production of alanine *via* transamination of glutamate (Fig. 6). The significantly higher lactate level in venous plasma (referred to as negative arteriovenous balance) is likely associated with its release to venous plasma as the end-product of pyruvate conversion. This observation is congruent with earlier findings on peripheral lactate levels^35^,^36^ and is representative of incomplete glycolysis even at rest, in aerobic conditions at early fasting metabolic state (Fig. 6). It also demonstrates the role of lactate as a mobile nutrient (gluconeogenic metabolite) that is further taken up by the liver to regenerate glucose and complete the Cori cycle^2^,^35^. The observations of a significantly lower levels of glutamate (positive AV balance), and increased levels of alanine (negative AV balance) and glutamine (negative AV balance) in venous plasma imply the uptake of glutamate (by muscle) and the production and release of alanine and glutamine by transamination. While early metabolic studies demonstrated the synthesis of glutamine and alanine, as two main gluconeogenic amino acids formed by transamination of α-ketoacids (pyruvate and oxaloacetate, Fig. 6) in muscle^8^,^37^, the uptake pattern of glutamate by human muscle has been reported only by Marliss *et al.* using enzymatic assays^38^. Our results reflect a significant uptake pattern of glutamate suggesting that glutamate is the major amino acid precursor, primarily as a nitrogen donor, in the synthesis of alanine and glutamine. Furthermore, a significant positive arteriovenous difference was observed for aspartate, suggesting that aspartate could play an important role in glutamate supply in the muscle cells. The uptake of aspartate by muscle is a new finding that is likely coupled to excess of α-ketoglutarate, produced by transamination of glutamate (Fig. 6). The excess of α-ketoglutarate could lead the amino transfer from aspartate to glutamate and concurrently replenish the TCA-cycle (Fig. 6). Early tracing experiments using rat muscle *in vitro* indicate that glutamate, aspartate and specific branched amino acids (isoleucine and valine) should enter the TCA cycle and serve as carbon skeleton and nitrogen supply for glutamine synthesis^7^,^8^, however only the uptake pattern of glutamate has been previously reported^38^, The uptake pattern of serine, reflected as lower concentration in venous blood, is a new finding as well, which may imply its usage for pyruvate anaplerosis, as a non-carbohydrate precursor, by serine dehydratase mediated direct conversion to pyruvate (Fig. 6). This finding supports the original formulation of the glucose-alanine cycle after which pyruvate is derived from glucose as well as other metabolized amino acids^37^. Tracer experiments in humans *in vivo* have implied that the carbon atoms of alanine are mostly derived from plasma glucose and muscle glycogen^8^,^39^ and that glutamine should be a major gluconeogenic precursor for a positive transfer of new carbon to the glucose pool^7^,^8^, originating primarily from protein-derived amino acids. Therefore, the hypothesis of serine deamination for pyruvate anaplerosis needs to be further investigated.

Although substantial release of free amino acids from muscle tissue (reflecting the muscle protein undergoing degradation) after an overnight fast has been reported in early studies^5^,^34^, and we did observe the negative arteriovenous differences for most of the quantified amino acids, only the release of alanine, glutamine and phenylalanine were observed at statistically significant levels. This implies that specific biochemical pathways that support primarily gluconeogenic metabolite synthesis (lactate, alanine, glutamine and phenylalanine) are dominant in human postabsorptive metabolic state. Overall, our results suggest that glutamate and aspartate are metabolized by muscle as primary nitrogen and/or carbon donors in the synthesis of other gluconeogenic metabolites, as demonstrated by their highly significant positive arteriovenous balance. The increased levels of TCA-cycle intermediates (e.g. succinate, malate, Figs 4 and 5) in venous plasma suggest that the metabolized amino acids are converted into TCA cycle intermediates that can be released to venous blood and/or used as carbon source mainly for the synthesis of glutamine^8^,^9^. Alanine seems to be directly produced by transamination of pyruvate, using the “uptaken” glutamate as nitrogen donor^39^. These observations affirm that muscle is a key gluconeogenic site in the body and provide some new, potentially physiologically important insights into its metabolic activity regarding the unanticipated role of aspartate and serine, related to the inter-organ carbon and nitrogen transfer *via* the Cori and analogous Glucose-Alanine cycle, to meet the energy needs of different cell types.

Beyond the above mentioned arteriovenous differences related to muscle metabolism, distinct metabolites, including oxidized fatty acids (HPODE family) and sialic acid may reflect the oxidation state difference between the arterial and venous plasma^40^,^41^,^42^,^43^,^44^,^45^. Surprisingly, HPODEs level in venous plasma was significantly higher compared to arterial plasma. It is noteworthy that HPODEs, the products of reactive oxygen species (ROS)-mediated peroxidation of linoleates and the primary products of the autoxidation process, are considered as potential biomarkers for oxidative stress status *in vivo*^43^,^44^. Our results may imply that the venous system is under an increased oxidative stress state when compared to the arterial system. The observed difference may also be the reflection of the peroxided lipid production in the muscle or capillary system. Lipid peroxidation is implicated in the pathogenesis of several diseases^44^,^45^, however its relation to venous disease has not yet been demonstrated. The arteriovenous oxidation state, with the focus on venous system, is a separate subject that demands further investigation.

The value of untargeted, paired arterial and venous plasma profiling is in the ability to examine very subtle changes related to tissue specific metabolism. As demonstrated here on human peripheral tissue, the finely tuned arteriovenous metabolite changes provided information on the active metabolic pathways in muscle, associated with an overnight fast. This information, which reflects the metabolic activity of an organ related to characteristic physiological state, would not be possible to obtain from the analysis of venous plasma alone. The observed differences highlight the roles of glutamate, lactate, glutamine and alanine, and the unanticipated roles of aspartate, serine and succinate as carbon and nitrogen shuttles in the body mainly *via* Cori and Glucose-Alanine cycle. Therefore, arteriovenous plasma profiling can elucidate new, functionally related metabolites based on patterns of metabolite transport at a specific level of circulatory system. The metabolite transport analysis further provide relevant information regarding the effects of various factors that influence nutrient uptake and/or assimilation and thus aid in defining normal organ homeostasis *vs.* specific organ pathology. The arteriovenous blood metabolite levels were also examined in relation to the clinical characteristics of the patients (e.g. gender, age, body mass index, Supplemental Table S2, Figure S3). However, to draw a meaningful conclusion related to the impact of specific clinical factors on a human blood profile the population cohort size should be larger.

Furthermore, the application of this approach would be especially useful when attempting to examine a cohort of individuals for changes in blood metabolite levels in relation to an organ specific disease (such as liver cirrhosis, or heart disease). Human populations have inherent inter-individual variation, and most studies require numbers in the hundreds if not thousands to identify a statistically significant disease biomarker in venous blood. Removing this variation through calculation of the paired arterial to venous blood ratios can reveal subtle biochemical changes impossible to pick up through large-scale screening of venous blood samples. We have already shown in the relatively small sample set here that these metabolite A/V ratios are stable between individuals; any large deviations from these ratios can thus alert us to possible metabolic pathway disruption. Therefore we propose that this approach will provide a new metabolic profiling model for blood analysis.

## Methods

### Sample preparation

A total of 20 normal, healthy subjects (10 males and 10 females) were recruited from the Normal Blood Donor Program and from the general population of The Scripps Research Institute employees. The subjects were screened by age (18 lower limit), body mass index (18–31) and applying the following clinical criteria: no hormone contraceptives, no topical estrogen agents, no statins/lipid lowering agents, no allergy medications at least 2 weeks prior to blood drawing, no diabetic medications, no anti-inflammatory medications at least 2 weeks prior to blood drawing, no heart failure medications, no anti-hypertensive medications and no anticoagulants. The blood was sampled after an overnight fast, assuring that all the subjects are in the same basal, steady metabolic state.

The arterial and venous bloods were sampled back to back, within 10 minutes, by a Respiratory Therapist and a Phlebotomist, respectively. The radial artery and the brachial vein approaches were used. A total of 3 mL of each blood were drawn using the Marquest™ Gaslyte® blood sampler with lyophilized lithium heparin as an anticoagulant. The samples were immediately transferred from the heparin syringe into labeled aliquot tube and centrifuged at 4 °C at 2000 rpm for 20 min. Then the plasma was aliquoted (0.5 ml) into appropriately labeled cryovial tubes and frozen −80 °C.

All experimental protocols were performed in accordance with the guidelines and regulations approved by the Scripps Institutional Review Board. All subjects have given their written informed consent.

### Metabolite extraction

Human plasma samples (100 μL) were extracted using 400 μL of cold MeOH:ACN (1:1, v/v) to maintain MeOH:ACN:H_2_O (2:2:1, v/v) ratio. The samples were then vortexed for 30 s. To precipitate proteins, the samples were incubated for 1 h at −20 °C, followed by 15 min centrifugation at 13,000 rpm and 4 °C. The resulting supernatant was removed and evaporated to dryness in a vacuum concentrator. The dry extracts were then reconstituted in 100 μL of ACN:H_2_O (1:1, v/v), sonicated for 3 min and centrifuged 10 min at 13000 rpm and 4 °C to remove insoluble debris. The supernatants were transferred to HPLC vials and stored at −80 °C prior to LC/MS analysis.

### Untargeted LC/MS analysis

Analyses were performed using an HPLC system (1200 series, Agilent Technologies, Santa Clara, CA) coupled to a 6538 UHD quadrupole time-of-flight (Q-TOF, Agilent Technologies). Samples were analyzed using Luna Aminopropyl, 3 μm, 150 mm x 1.0 mm I.D. column (Phenomenex, PA) for HILIC/MS analysis. For HILIC in ESI negative mode, the mobile phase was composed of A = 20 mM ammonium acetate and 20 mM ammonium hydroxide in 95% water and B = 95% acetonitrile. The linear gradient elution from 100% B (0–5 min) to 100% A (50–55 min) was applied. The 10 minutes post-run was applied for HILIC, to insure the column re-equilibration and to maintain the reproducibility. The flow rate was 50 μL/min and the sample injection volume was 8 μL. ESI source conditions were set as following: gas temperature 325 °C, drying gas 5 L/min, nebulizer 15 psi, fragmentor 120 V, skimmer 65 V, and capillary voltage 4000 V in ESI negative mode. The instrument was set to acquire over the *m/z* range 60-1000, with the MS acquisition rate of 1.67 spectra/s. For the MS/MS of selected precursors the default isolation width was set as medium (4 Da), with a MS acquisition rate at 1.67 spectra/s and MS/MS acquisition at 1.67 spectra/s. The collision energy was fixed at 20 V.

Additional LC/MS/MS analyses were performed using an HPLC system (1200 series, Agilent Technologies) coupled to TripleTOF 5600 (Q-TOF, AB Sciex). The TripleTOF mass spectrometer was used for its ability to acquire MS/MS spectra on an information-dependent basis (IDA) during an LC/MS experiment. In this mode, the acquisition software continuously evaluates the full scan survey MS data as it collects and triggers the acquisition of MS/MS spectra depending on preselected criteria. In each cycle, 15 precursor ions were chosen for fragmentation at collision energy (CE) of 30 V (15 MS/MS events with product ion accumulation time of 50 msec each)^46^. ESI source conditions were set as following: Ion source gas 1 as 15, Ion source gas 2 as 10, Curtain gas as 10, source temperature 550 °C, Ion Spray Voltage Floating (ISVF) 5500 V or −4500 V in positive or negative modes, respectively. LC conditions (column, mobile phase and gradient) were the same as described for HILIC untargeted profiling.

### Targeted LC/MS analysis

Quantitation of metabolites of interest was performed using an HPLC system (1200 series, Agilent Technologies) coupled to Triple quadrupole 6450 (QqQ, Agilent) mass spectrometer. It was operated in Dynamic multiple reaction monitoring mode (MRM), where the collision energies and product ions (MS2 or quantifier and qualifier ion transitions) were pre-optimized for each metabolite of interest (Table 1). Cycle time was 500 ms. ESI source conditions were set as following: gas temperature 325 °C, drying gas 8 L/min, nebulizer 30 psi and capillary voltage 3500 V in ESI positive and negative mode. The analyses were performed in ESI negative mode using the identical Phenomenex aminopropyl column as for untargeted analysis, with the same mobile phases. Metabolites were quantified in three different batches, first batch (metabolites labeled by numbers from 1 to 12 and 15 to 17, Table 1) was analyzed in a positive ionization mode, using the gradient from 80% B (0–5 min) to 50% B (15 min); second batch (metabolites labeled by numbers 13 and 14, Table 1) was analyzed in a negative ionization mode, using the gradient from 50% B (0–2 min) to 0% B (7–12 min) and third batch (metabolites labeled by numbers from 18 to 36, Table 1) was analyzed in negative ionization mode, using the gradient from gradient 100% B (0–5 min) to 70% A (40 min). The 10 minutes post-run was applied to insure the reproducibility. The injection volume was 3 μL for all analyzed plasma extracts. Standard compound mixtures were used for method optimization and calibration.

### Quality control assay

A YSI 2900 Bioanalyzer was used to determine the absolute concentration of glutamine, glutamate, glucose, and lactate in plasma, according to manufacturer’s instructions. Linearity curves were generated for each analyte using calibration solution and the integrity of the system was confirmed by daily integrity tests. 25 uL of plasma was used per measurement and glutamine/glutamate and glucose/lactate were measured concomitantly.

### Chemometric analysis

The raw LC/MS data were converted to mzXML files using ProteoWizard MS Convert version 3.0.4146. The mzXML files were processed using XCMS online software for peak detection, chromatogram alignment and isotope annotation^10^,^47^. The parameters in XCMS were set as follows: centWave settings for feature detection (Δ *m/z* = 15 ppm, minimum peak width = 10 seconds and maximum peak width = 120 seconds); obiwarp settings for retention time correction (profStep = 1); and parameters including mzwid = 0.015, minfrac = 0.5 and bw = 5 for chromatogram alignment. Peak area was used for semi-quantification in untargeted profiling. Differentially expressed metabolite features were filtered out using statistical treshold parameters: p-value ≤ 0.01, Intensity (ion counts) >10,000 and median fold change ≥1.3. Metabolites were selected for the identification after filtering out the isotopes, adducts, in-source fragments, multiple charged species features and features whose identity remained unresolved due to the absence of hits in currently available databases (METLIN, HMDB, Lipid maps).

### Metabolite identification

Statistically significant differentially expressed features in arterial *vs.* venous plasma were identified by accurate mass (mass error < 5 ppm), MS/MS fragmentation pattern and retention time. Accurate masses were searched against databases METLIN and HMDB. The identifications were made by matching the acquired MS/MS data for metabolites of interest in plasma extract against MS/MS data recorded for standards, assembled in in-house developed and online available METLIN database^11^,^27^. The identifications were complemented with retention time information obtained from LC/MS analysis of standard compounds performed in identical conditions.

### Metabolite ratio quantification

The ion response for each standard solution was determined by integrating the area of the quantifier transitions listed in Table 1 for each compound (Agilent QQQ Quantitative Analysis). Standard curves were then constructed by plotting ion response vs. concentration injected into the QqQ mass spectrometer, on the basis of 6 dilutions (by factor 2) and three replicates of each dilution. Physiological concentrations of metabolites in arterial and venous plasma were not reported because the absolute quantification should be performed using the stable isotope-labeled standards. Therefore, we have reported the validated peak area response and metabolite ratios.

### Statistical analysis

In both, untargeted and targeted validation approach, the non-parametric, two-tailed Wilcoxon matched pairs signed rank test was used to assign for statistically significant, differentially expressed features in arterial *vs.* venous plasma. The Wilcoxon rank test was used as an alternative to paired parametric t-test because the assumptions of normality (Shapiro-Wilk test) and homosedacity (Levene test) were not met.

## Additional Information

**How to cite this article**: Ivanisevic, J. *et al.* Arteriovenous Blood Metabolomics: A Readout of Intra-Tissue Metabostasis. *Sci. Rep.* **5**, 12757; doi: 10.1038/srep12757 (2015).

## Supplementary Material

Supplementary Information

## Figures and Tables

**Table 1:** List of quantified metabolites with matching precursor ions, [M-H]- in negative (13–14, 18–36) and [M + H]+ in positive ionization mode (1–12, 15–17), optimized transition states (quantifier and qualifier ions), fragmentor and collision energies. Metabolite numbers match the list in Fig. 4.

	Metabolite	Precursor ion (MS1)	Quantifier ion transition (MS2)	Qualifier ion transition(s) (MS2)	Fragmentor (V)	Collision energy (V)
**1**	Leucine	132.1	44.1	86.1	65	23/7
**2**	Isoleucine	132.1	69.1	86.1	56	19/15
**3**	Valine	118.1	72.1	55.1	53	7/19
**4**	Methionine	150.1	104.0	56.1	77	7/31
**5**	Proline	116.1	70.1	43.1	86	15/43
**6**	Alanine	90.1	44.1	44.1	35	8
**7**	Tyrosine	182.1	136.1	91.1	74	11/27
**8**	Glycine	76	30.0	30.0	75	5
**9**	Threonine	120.1	56.1	74.1	56	15/7
**10**	Asparagine	133.1	74.0	46.0	71	11/15
**11**	Serine	106.1	60.1	42.1	50	7/27
**12**	Histidine	156.1	56.1	110.1	92	39/27
**13**	Malate	133	115	71	50	10
**14**	α-ketoglutarate	145	101	57	40	5
**15**	Arginine	175.1	70.1	70.1	95	23
**16**	Ornithine	133.1	70.1	116.1	62	15/7
**17**	Lysine	147.1	84.1	130.1	77	15/7
**18**	Allantoin	157.0	97.0	114.0	70	9/9
**19**	Docosahexaenoic acid	327.2	283.3	229.2/177.2/59.1	132	5/9/9/21
**20**	Arachidonic acid	303.2	259.3	285.2/205.2/59.1	129	9/9/9/17
**21**	Hypoxanthine	135.0	92.0	65.0	111	13/29
**22**	Phenylalanine	164.1	147.1	103.1	62	9/13
**23**	Tryptophan	203.1	116.1	159.1	108	9/5
**24**	Inosine	267.1	135.0	108.0	129	17/37
**25**	Hippuric acid	178.1	77.1	134.1	62	13/9
**26**	Glutamine	145.1	127.1	109.1	62	9/9
**27**	Lactate	89.0	43.1	43.1	55	12
**28**	Sialic acid (Neu5Ac)	308.1	87.0	170.1	111	9/9
**29**	Aspartate	132.0	88.1	115.0	62	9/9
**30**	Glutamate	146.0	102.1	128.0	62	9/9
**31**	Cystine	239.0	120.0	74.0	62	5/21
**32**	Hexadecanedioic acid	285.2	267.2	223.2	129	13/13
**33**	Tetradecanedioic acid	257.2	239.2	195.2	126	9/13
**34**	Dodecanedioic acid	229.1	211.1	167.1	120	9/13
**35**	3-methylglutaric acid	145.1	101.1	83.0	59	9/25
**36**	Succinate	117.0	73.0	73.0	62	9
