# Supplementary material for: Arteriovenous Blood Metabolomics: A Readout of Intra-Tissue Metabostasis
Source: Sci Rep. 2015 Aug 5;5:12757. doi: 10.1038/srep12757 (PMC4525490; doi:10.1038/srep12757)
Supplement: Supplementary Information [file srep12757-s1.pdf]

Supplemental Information for Publication

Arteriovenous Blood Metabolomics: A Readout of Intra-Tissue Metabostasis

Julijana Ivanisevic[1], Darlene Elias[2,3], Hiroshi Deguchi[2], Patricia M. Averell[3], Michael Kurczy[1], Caroline H. Johnson[1], Ralf Tautenhahn[1], Zhengjiang Zhu[1], Jeramie Watrous[4], Mohit Jain[4], John Griffin[2], Gary J. Patti[5] and Gary Siuzdak[1,6]

Table S1. List of putatively identified metabolites whose levels were varying in arterial *vs.* venous human plasma as revealed by untargeted metabolic profiling. Metabolites were putatively identified by the exact mass and specific MS/MS fragments. *No MS/MS match in current databases (METLIN, HMDB)

Observed m/z	Putative ID	Exact mass (ppm error)	Up-regulation	p-value	Median fold change
[M-H]- 146.0464	Glutamic acid	147.0532(3 ppm)	Arterial plasma	3.8E-06	2.6
[2M+Na-2H]- 201.0388	Lactic acid	90.0317(5 ppm)	Venous plasma	4.2E-04	1.4
[M-H]- 327.2334	Docosahexaenoic acid (DHA)	328.2406(1 ppm)	Arterial plasma	3.92E-03	1.7
[M-H]- 303.2333	Arachidonic acid	304.2402 (1 ppm)	Arterial plasma	4.58E-03	1.3
[M-H]- 135.0319	Hypoxanthine	136.0385 (4 ppm)	Venous plasma	8.23E-03	1.3
[M-H]- 239.0166	Cystine	240.0239 (0 ppm)	Venous plasma	2.70E-04	1.3
[M-H]- 179.0577	Paraxanthine	180.0647 (2 ppm)	Arterial plasma	1.2E-02	1.3
[M-H]- 157.0372	Allantoin	158.0440 (3 ppm)	Arterial plasma	3.3E-03	1.4
[M-H]- 178.0515	Hippuric acid	179.0582 (3 ppm)	Arterial plasma	9.45E-03	1.5
[M-H]- 285.2069	Hexadecanedioic acid	286.2144 (1 ppm)	Venous plasma	8.23E-03	5.7
[M-H]- 257.1754	Tetradecanedioic acid	258.1831 (2 ppm)	Arterial plasma	7.629E-05	1.9
[M-H]- 229.1442	Dodecanedioic acid	230.1518 (1 ppm)	Venous plasma	4.58E-03	1.2
[M-H]- 308.0987	N-Acetylneuraminic acid	308.0987 (0 ppm)	Venous plasma	1.0E-04	3

[M-H]- 311.2224	Hydroxy Unsaturated Fatty acids, Hydroxyperoxy-octadecadienoic acid variant*	312.2301 (1 ppm)	Venous plasma	9.7E-04	34.8
[M-H]- 309.2067	Hydroxy Unsaturated Fatty Acids, Hydroperoxy-octadecatrienoic acid variant*	310.2144 (1 ppm)	Venous plasma	4.2E-04	32.1

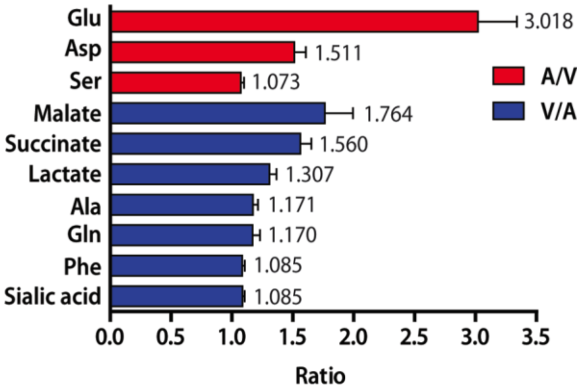

Figure S1. Bar graphs displaying A/V and V/A ratios of metabolites that were significantly decreased and increased in venous *vs.* arterial plasma. Mean ratios are indicated above the error bars (SEM).

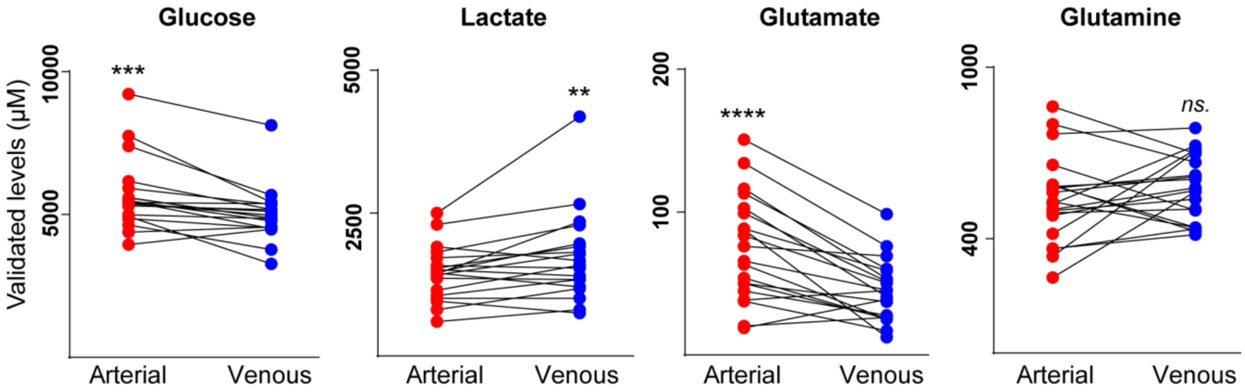

Figure S2. Metabolites whose levels were measured using Bioanalyzer assay. Paired plots show the absolute concentrations of each metabolite in arterial and venous plasma of each individual. Significance level of Wilcoxon test is indicated by the number of stars.

Table S2. Population cohort description using the age, weight, BMI and gender of each individual.

PtID #	Age	Gender	Height	Weight	BMI	BMI category*	Age category
**1**	50	M	71	195	27.2	Overweight	45-55
**2**	51	M	65	180	30	Obesity	45-55
**3**	41	M	74	209	26.8	Overweight	35-45
**4**	23	M	74	175	22.5	Normal weight	15-25
**5**	56	F	65	172	28.6	Overweight	55-65
**6**	33	M	71	175	24.4	Normal weight	25-35
**7**	31	M	70	170	24.4	Normal weight	25-35
**8**	51	F	66	178	28.7	Overweight	45-55
**9**	32	M	67	158	24.7	Normal weight	25-35
**10**	40	M	71	216	30.1	Obesity	35-45
**11**	41	F	63	120	21.3	Normal weight	35-45
**12**	62	F	64	165	28.3	Overweight	55-65
**13**	17	F	/	/	/	/	15-25
**14**	39	M	73	195	25.7	Overweight	35-45
**15**	31	F	70	190	27.3	Overweight	25-35
**16**	26	F	63	120	21.3	Normal weight	25-35
**17**	46	F	67	135	21.1	Normal weight	45-55
**18**	27	F	63	180	31.9	Obesity	25-35
**19**	41	F	59	129	26.1	Overweight	35-45
**20**	33	M	72	185	25.1	Overweight	25-35

***LEGEND**

Underweight = <18.5

Normal weight = 18.5–24.9

Overweight = 25–29.9

Obesity = BMI of 30 or greater

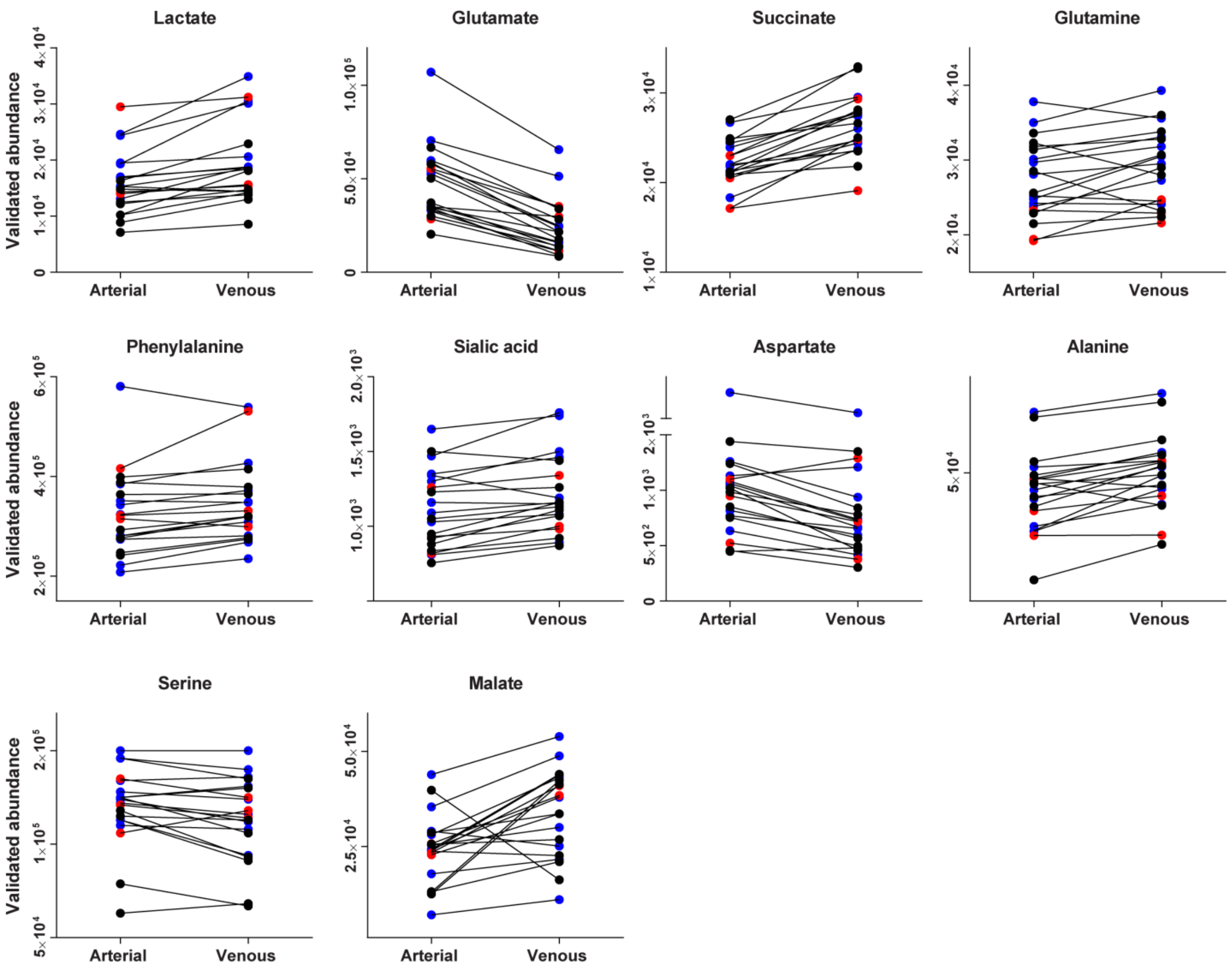

Figure S3. Abundances of metabolites in arterial and venous blood depending on a Body Mass Index (BMI) of each individual. Paired plots show the abundances of each metabolite in arterial and venous plasma of each individual. Red dots - obese individuals, Blue dots – overweight individuals, Black dots – normal weight individuals
